# A phase Ia/Ib clinical trial of metronomic chemotherapy based on a mathematical model of oral vinorelbine in metastatic non-small cell lung cancer and malignant pleural mesothelioma: rationale and study protocol

**DOI:** 10.1186/s12885-016-2308-z

**Published:** 2016-04-20

**Authors:** Xavier Elharrar, Dominique Barbolosi, Joseph Ciccolini, Christophe Meille, Christian Faivre, Bruno Lacarelle, Nicolas André, Fabrice Barlesi

**Affiliations:** Multidisciplinary Oncology and Therapeutic Innovations department, Assistance Publique Hôpitaux de Marseille, Aix Marseille University, Marseille, France; SMARTc Pharmacokinetics Unit, Aix Marseille University, Inserm S_911 CRO2, Marseille, France; Assistance Publique Hôpitaux de Marseille, Centre d’Essais Précoces en Cancérologie de Marseille APHM CLIP2, Aix Marseille University, Marseille, France; Paediatry Oncology Unit, Assistance Publique Hôpitaux de Marseille, Marseille, France

**Keywords:** Lung cancer, Mesothelioma, Metronomics, Vinorelbine, Modelling and simulation, Computational oncology

## Abstract

**Background:**

Metronomic oral vinorelbine is effective in metastatic NSCLC and malignant pleural mesothelioma, but all the studies published thus far were based upon a variety of empirical and possibly suboptimal schedules, with inconsistent results. Mathematical modelling showed by simulation that a new metronomic protocol could lead to a better safety and efficacy profile.

**Design:**

This phase Ia/Ib trial was designed to confirm safety (phase Ia) and evaluate efficacy (phase Ib) of a new metronomic oral vinorelbine schedule. Patients with metastatic NSCLC or malignant pleural mesothelioma in whom standard treatments failed and who exhibited ECOG performance status 0–2 and adequate organ function will be eligible. Our mathematical PK-PD model suggested an alternative weekly D1, D2 and D4 schedule (named Vinorelbine Theoretical Protocol) with a respective dose of 60, 30 and 60 mg. Trial recruitment will be two-staged, as 12 patients are planned to participate in phase Ia to confirm safety and consolidate the calibration of the model parameters. Depending on the phase Ia results and after a favourable decision from a consultative committee, the extension phase (phase Ib) will be an efficacy study including 20 patients who will receive the Optimal Vinorelbine Theoretical Protocol. The primary endpoint is the tolerance (assessed by CTC v4.0) for the phase Ia and the objective response according to RECIST 1.1 for phase Ib.

An ancillary study on circulating angiogenesis biomarkers will be a subproject of the trial.

**Discussion:**

This ongoing trial is the first to prospectively test a mathematically optimized schedule in metronomic chemotherapy. As such, this trial can be considered as a proof-of-concept study demonstrating the feasibility to run a computational-driven protocol to ensure an optimal efficacy/toxicity balance in patients with cancer.

**Trial registration:**

EudraCT N°: 2015-000138-31

## Background

The management of cancer has evolved towards more personalized treatment, based particularly on the use of bio-guided treatments [1]. Nevertheless, all types of lung cancer are not eligible for these treatments and chemotherapy remains a standard treatment in and after the first-line for many patients. With the improvement of palliative care, the development of new treatments beyond first-line in metastatic NSCLC contributed to the increase in overall survival. According to American Society of Clinical Oncology (ASCO) [2] and French guidelines, three treatments may be proposed in second-line: docetaxel, pemetrexed and erlotinib. One is available in third-line therapy: erlotinib. Regarding malignant pleural mesothelioma (MPM), there is to date no standard treatment beyond first-line chemotherapy. After validated treatments, standard management is based on palliative care. However, some chemotherapy can be active in these diseases and thus can be used apart from guidelines for patients with adequate functional status. In these situations, prescription is based on empirical dosing regimens, and therefore can be probably optimized.

The conventional approach with chemotherapeutic drugs attempts to use a dose close to the Maximum Tolerated Dose (MTD) to maximize efficacy. Depending on the drug, administration can be followed by a rest period for the recovery of healthy tissues. This period without treatment, required after a high cytotoxic dose, can promote tumour repopulation and the emergence of resistant clones. In addition, the role of angiogenesis in tumour growth and metastatic spreading and the discovery of anti-angiogenic properties of some chemotherapies, including vinorelbine, have caused the recent development of strategies, called metronomic [3, 4] chemotherapy. The anti-angiogenic efficacy of chemotherapy seems to be optimized by administering comparatively low doses of drugs on a frequent or continuous schedule, with no extended interruptions [5]. Metronomic chemotherapy involves several mechanisms of action. Besides the direct effect of the cytotoxic effect on cancerous cells, it allows for the inhibition of endothelial proliferation and possibly a weakening of the immune response [6].

### Vinorelbine

Vinorelbine is a semi-synthetic vinca-alkaloid that acts by inhibiting tubulin polymerization during mitosis. It is as active on the mitotic microtubules as other vinca-alkaloids, but less active against axonal microtubules, thus explaining its lower neurotoxicity [7].

Vinorelbine can be administered either intravenously or orally. The bioavailability of oral vinorelbine varies from 33 to 43 % [8, 9]. The tolerance is well documented and is comparable to the intravenous form [7].

The therapeutic efficacy has been reported clinically and might legitimize the use of vinorelbine in patients with lung cancer. However, this drug is also associated with a significant risk of haematological and non-haematological adverse events in these fragile and pre-treated patients [10].

Many features of vinorelbine make it an ideal candidate for the development of metronomic strategies. Vinca alkaloids, including vinorelbine, have an anti-angiogenic activity, demonstrated in vitro [11, 12] and in vivo [13–28] after metronomic dosing. Due to its ease of administration, oral vinorelbine has paved the way for innovative treatment strategies through metronomic regimens.

### Vinorelbine in metastatic NSCLC

In the first-line setting for advanced NSCLC, three phase III trials have investigated the combination of vinorelbine with platinum derivatives [13–15]. These studies confirmed the superiority of a combination of platinum derivatives and vinorelbine over single-agent chemotherapy. Subsequently, several meta-analyses [16–18] have demonstrated that the use of vinorelbine-based regimens may be less effective in controlling disease than other combinations with platinum derivatives in first-line therapy. Similarly, the use of single-agent vinorelbine as first-line therapy for advanced disease in fit elderly patients has been supplanted by the carboplatine/weekly paclitaxel doublet [19].

In the second-line setting, vinorelbine was one of the comparators (along with ifosfamide) of a phase III trial of second line docetaxel [20] in which docetaxel (75 mg/m2) led to better overall survival than the comparators, thus establishing it as the standard choice for second-line treatment. A retrospective study that evaluated 39 patients with stage IIIb or IV treated with vinorelbine after a median of two prior lines of treatment, showed a partial response rate of 7.7 % and stable disease in 25.6 % of patients [21].

However, several clinical trials have evaluated vinorelbine in metronomic regimens in different tumour types, including NSCLC [22–24]. In a first metronomic clinical trial, conducted in 62 patients with advanced cancers, vinorelbine has been tested at doses ranging from 20 to 70 mg thrice a week [22]. At the end of this phase IA, the preferred effective dose was 50 mg thrice a week. Afterward, a phase IB trial, led by the same team in 101 patients with breast cancer, prostate cancer or NSCLC, compared the following dosages: 30 mg thrice a week, 40 mg thrice a week and 50 mg thrice a week. Despite the differences in dose, progression-free survival and tolerance did not differ in the 3 groups, with a total of 10 % of grade 3–4 neutropenia [23]. A metronomic chemotherapy Phase II trial of oral vinorelbine 50 mg thrice a week was conducted in 46 patients with treatment failure in the treatment of NSCLC. The response rate was 10.9 %. More side effects were recorded in this study, with mostly grade 3–4 neutropenia in 23.9 % of patients and febrile neutropenia in 10.9 % of them [24]. Recently, a prospective study recruited 43 chemotherapy naive elderly patients with stage IIIB-IV NSCLC, treated with oral vinorelbine 50 mg thrice a week. Overall response rate (ORR) was 18.6 % and tolerance was at an acceptable level [25].

### Vinorelbine in malignant pleural mesothelioma

Vinorelbine has demonstrated activity in MPM. The European Society for Medical Oncology (ESMO) guidelines consider that vinorelbine might be a reasonable choice in second-line therapy [26]. A non-comparative phase II study in which vinorelbine was administered at a dose of 30 mg/m^2^ for 6 weeks in 63 patients previously treated with chemotherapy found a 16 % response rate and controlled disease in 68 % of patients. A major haematologic toxicity was noted, with a grade 3–4 neutropenia in 55 % of patients [27]. These second-line response rates were confirmed by a recent retrospective study on 59 patients included after a first-line treatment, including pemetrexed. The response rate was 15.2 %, with a 49.1 % disease control rate and a 6.2-month overall survival. In this study, vinorelbine, administered at a dose of 25 mg/m^2^ D1-D8 every three weeks, had a better tolerance with grade 3–4 neutropenia in 8.4 % of patients [28].

### Pharmacokinetic-pharmacodynamic modelling

These clinical trials showed the limits of empirical approaches to determine appropriate metronomic strategies in oncology. These first results, obtained in different populations and with empirical treatment regimens, suggest the development of new approaches, such as bio-mathematical modelling, for the selection of metronomic schedules. Complex situations inherent to the administration of anticancer agents can be managed using pharmacokinetic-pharmacodynamic models (PK-PD). Previous studies showed that numbers of critical questions have to be addressed when switching to a metronomic dosing regimen. Which dose should be used? What is the best dosing schedule? Among the most effective protocols, which one will be the least toxic? Because countless schedules are possible, testing them empirically, either clinically or using non-clinical models, seems to be an unachievable goal. Mathematical modelling can be an effective tool to address the above questions [29]. The modelling approach can transpose into mathematical language the action of the drug on the body, both for efficacy and toxicity. Once the model is built, a search for an optimal solution (i.e., protocol achieving greater efficacy while respecting pre-defined constraints on toxicity) can be performed in silico by the iterative simulation of thousands of different schedules. Studies have provided further support for this method [30].

### Objectives

The aim of the clinical trial presented below is to effectively use oral vinorelbine in a metronomic schedule, developed and validated by mathematical modelling, in patients with NSCLC or MPM after failure of standard treatment.

## Methods

### Study design

This study is a prospective non-randomized phase Ia/Ib trial of the single agent oral metronomic vinorelbine. The study will take place in the Phase I Oncology Unit (Centre d’Essais Précoces en Cancérologie de Marseille APHM CLIP^2^), 264 rue Saint-Pierre, 13005 Marseille, France.

- Phase Ia: Validation of the simulated theoretical dosing schedule (Vinorelbine Theoretical Protocol) and enrichment of model data.
- Phase Ib: Expansion phase: proof of concept and efficacy study to be started after a favourable decision by the study monitoring committee.

### Mathematical modelling

Several model-based strategies to address the issue of keeping drug-related toxicities under control while improving anti-tumour efficacy [31–33] have been developed. Recently, our group has developed a PK-PD mathematical model specifically dedicated to the management of oral metronomic chemotherapy [29, 34]. The model is divided into 3 parts:

- Pharmacokinetics: This model [35] describes the evolution over time of blood concentrations of the drug. The PK model is a linear 3 compartmental model. Absorption, bioavailability, distribution and elimination processes are quantified by pharmacokinetic parameters. These parameters can be individualized for each patient, e.g., using the Bayesian method with patient drug concentration measurement. PK profile will be considered as known for PD simulations.
- Pharmacodynamic toxicity: This model describes the impact of the drug concentration on haematopoietic cells [36], resulting in neutropenia for vinorelbine. This model required the adaptation of the work of Friberg [37] due to the continuous dosing associated with the metronomic dosing regimen. Model simulations integrate inter-individual variability on baseline value, maturation time and regulation process.
- Pharmacodynamic efficacy: This model describes the action of the drug on both tumour cells and endothelial cells, as well as the emergence of resistant clones induced by the treatment. Model parameters can be adjusted based on observed data, including inter-individual variability on initial tumour mass.

**Fig. 1:**
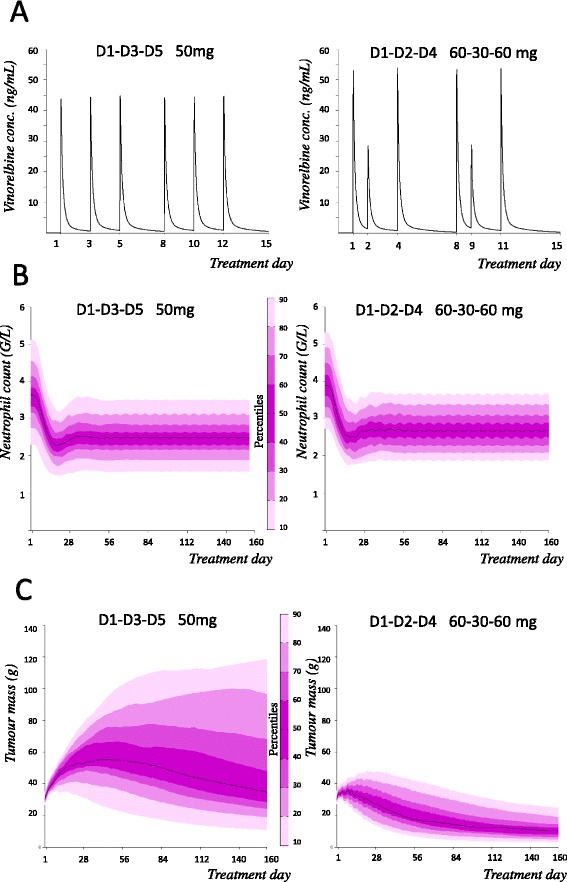
PK and PD simulation of two metronomic protocols. Comparative model prediction for a median PK (**a**) profile, neutrophil count (**b**) and tumor weight (**c**) profiles with inter-individual variability, for 160 days, of two different metronomic protocols: Briasoulis et al. schedule (i.e., 50 mg on D1, D3, D5) vs the computational-based schedule Vinorelbine Theoretical Protocol (i.e.,60, 30, 60 mg on D1, D2, D4). Whereas similar tolerance is predicted between both schedules, the VTP schedule could lead to a much greater impact on tumor growth, with less variability

With adjusted parameters, the PK-PD model described adequately the clinical data published by Briasoulis et al. (30, 40 and 50 mg, thrice a week, D1, D3, D5) both in terms of efficacy and toxicity. In a second step, the model was used to simulate, for a known average PK profile, alternate continuous metronomic schedules, achieving higher efficacy while being tolerated. A dosing regimen weekly D1, D2 and D4 with 60, 30 and 60 mg dose respectively was selected. Simulation of this protocol, named VTP (Vinorelbine Theoretical Protocol), resulted in more favourable efficacy profile and significant reduction of variability in response while maintaining a total dose per week of 150 mg (Fig. 1).

### Patients

To be eligible for inclusion, patients will have to provide signed informed consent forms before undergoing any study-related procedures and have the willingness and ability to comply with scheduled visits, laboratory tests and other study procedures. All patients must be aged 18 or older, have histologically or cytologically proven metastatic NSCLC or MPM, and a progression of disease after standard treatments. All patients must have at least one measurable target by RECIST 1.1. and an Eastern Cooperative Oncology Group (ECOG) performance status between 0 and 2. An adequate haematological (neutrophil count ≥ 1,500/mm^3^, platelet count ≥ 100,000/mm^3^, haemoglobin ≥ 9.0 g/dL), hepatic (total bilirubin ≤ 1.5 x ULN, aspartate transaminase (AST) and alanine transaminase (ALT) ≤ 2.5 x upper limit of normal (ULN), or AST and ALT ≤ 5 x ULN (if liver function abnormalities are due to cancer) and renal (creatinine clearance based on the Cockcroft-Gault formula ≥ 45 ml/min) function is required. Time between the end of the previous treatment and inclusion in the clinical study will have to be 4 weeks for experimental treatment as part of a trial, or 3 weeks for chemotherapy, or if this time is less than 4 weeks, 5 times the half-life of previous treatment for targeted therapy. Patients will be ineligible to participate if they have a peripheral neuropathy grade > 1, an uncontrolled cardiac disease requiring treatment (heart failure, angina pectoris, arrhythmia), a recent myocardial infarction (≤6 months) or a history of cancer other than basal cell carcinoma, treated cervical intraepithelial neoplasia or any other cancer treated without recurrence for at least 2 years. Patients with an active infection or any other serious medical or psychiatric condition that could affect participation in the trial (according to the opinion of the investigator) will also be excluded.

### Intervention

- Phase IA: patients will be enrolled and treated according to the schedule and the dose defined by the simulated theoretical model VTP. This Protocol (VTP) is based on oral vinorelbine 60 mg on day 1, 30 mg on day 2 and 60 mg on D4, weekly. In this step, data from PD efficacy (tumour assessments and monitoring of biological markers) will be collected and integrated to refine PD model parameters. Possibly, data from other ongoing trials of vinorelbine may be used. The optimal schedule (OVTP) will be simulated by the model. The final schedule will be affirmed after collegial consultation, taking into account biomathematics results, technical requirements and clinical constraints reviewed by oncologists and pharmacologists. From the VTP protocol, the OVTP could have modifications in the overall dose (which should not exceed 10 %) or in the administration schedule.
- Phase IB: Patients enrolled will receive vinorelbine according to the OVTP protocol obtained in the first step.

Patients will benefit all useful premedications and post-medications.

### Follow-up and duration of the study

During the continuation of protocol-defined treatment, laboratory tests (cell blood count) and clinical examination will be conducted at D1 of each week. Tumour response will be assessed at baseline and every 6 weeks from the 1^st^ day of treatment by performing a cerebral, thoracic, abdominal and pelvic CT scan. All assessments will be performed by investigators using RECIST version 1.1 (1.1 modified for MPM). The treatment will be administered until radiological disease progression, unacceptable toxicity, or withdrawal of consent.

After the end of the treatment period, patients will be seen every month or contacted by phone to collect data on potential adverse events and survival. The planned duration of the trial is estimated to be 36 months (duration of inclusions: 12 months; Follow-up: up to 24 months after the last patient inclusion).

### Outcomes and assessments

The primary endpoint of the phase Ia is the tolerance, assessed by CTC v4.0, with an objective of less than 10 % of patients with haematological toxicity (neutropenia) grade 3–4.

The primary endpoint of the phase Ib is the objective response according to RECIST 1.1 (NSCLC) or modified RECIST 1.1 (MPM).

Secondary endpoints include Overall Response Rate (ORR), Duration of Response (DR), Duration of Disease Control (DDC), Disease Control Rate (DCR), Progression-Free Survival (PFS), Overall Survival (OS), Tolerance (assessed according to CTC V4), Quality of life assessed by LCSS (Lung Cancer Symptom Scale) and EORTC QLQ C30 and C13, the PK-PD relationship of oral vinorelbine under VTP and OVTP schedules.

### Pharmacokinetics

Individual pharmacokinetic analysis will be based on two sets of four blood samples to determine the individual pharmacokinetic profiles of vinorelbine. Sampling times will be calculated by a D-optimality method based on literature data on the pharmacokinetics of vinorelbine [38].

### Circulating biomarkers

During this clinical trial, an ancillary study on biomarker analyses will be conducted: circulating endothelial cells (CEC), fibroblast growth factor 2 (FGF2), vascular endothelial growth factor (VEGF), interleukin-8 (IL-8), and thrombospondin-1 (TSP1) and Tie2/Tek. The results will be analysed to determine any correlation with the data obtained by RECIST in terms of tumour response and/or efficacy.

### Sample size determination and power

The expected number of patients is 32, with the following distribution:

- Phase Ia: 12 patients will receive VTP: the minimum sufficient number of patients to consolidate the calibration of the model parameters and define the optimal administration schedule (OVTP)
- Phase Ib: expansion phase after a favourable decision by the monitoring committee.

Twenty patients will receive the OVTP schedule.

The number of patients in the study is based on the Fleming one-step method, with a type I error (α) equal to 5 % and a type II (β) equal to 10 %.

The statistical hypotheses are determined by:

- p0: disease control rate that would be insufficient to continue with Phase II, set at 25 %;
- p1: minimum effective control rate to justify the continuation of the phase II trial, set at 50 %.

The following will be tested: H0: *p* = p0 ≤ 25 % versus H1: *p* ≥ p1 = 50 %. According to these hypotheses, the calculation shows that 20 patients should be included in the study.

### Statistical analyses

All patients receiving at least one dose of study drug will be included in the evaluation of safety. All patients who received at least one dose of study drug will be included in the intention-to-treat population and included in the assessment of effectiveness. Patients with valid pharmacokinetic data will be included in the pharmacokinetic analyses.

All efficacy parameters will be analysed descriptively. For the ORR and DCR, estimated 90 % and 95 % confidence intervals will be provided. For the PFS, the duration of the response and overall survival, the Kaplan-Meier method, the median durations and 95 % confidence intervals will be calculated.

### Monitoring committee

It is expected that a consultation meeting will be organized between the members of the project and the pharmaceutical company Pierre Fabre, after the inclusion of 12 patients in Phase I (4 weeks after the D1 of the 12^th^ included patient). This meeting will aim to analyse the adequacy of the model prediction and clinical outcomes (review of toxicity and clinical efficacy) in perspective with other results held by Pierre Fabre on trials underway elsewhere.

The transition to the second stage will take place after a favourable decision of the monitoring committee.

### Ethical approval

The study protocol was approved by the French regulatory authorities and by the appropriate French ethics committees (*Comité de protection des personnes (CPP) and Agence nationale de sécurité du médicament et des produit de santé (ANSM)*). The study was registered with EudraCT 2015-000138-31.

## Discussion

The ever-growing amount of knowledge on cancer biology and the systematic use of combined strategies for treating cancer patients make the optimization of currently available cancer therapies a challenging issue.

The metronomic scheduling applied to vinorelbine has generated inconsistent results in terms of efficacy and tolerance. Using a modelling approach is a new promising strategy to ensure safer and more efficient metronomic schedules in patients with cancer. When developing a metronomic regimen, such computational support could be particularly relevant, especially because several modalities can be considered and the selection cannot be done empirically. To date, only a few trials have been conducted with drug dosing regimen entirely driven by a mathematical model [39, 40]. To our knowledge, this is the first metronomic clinical study with a mathematically optimized schedule. Based on published data from previous vinorelbine studies, a PK-PD model was used to select a more efficient dosing regimen while keeping haematological toxicity at an acceptable level. This improved schedule is maintaining a same total dose per week than the established metronomic vinorelbine regimen from the previous studies [22–24].

The aims of this trial are to validate the tolerance of the improved metronomic VTP schedule in Phase IA and to evaluate the efficacy and safety of the final schedule OVTP in the phase IB in patients with NSCLC or MPM with treatment failure. Another objective of this trial is to perform an ancillary study with biomarker analysis. Using these biomarkers was justified by the results obtained in previous clinical studies integrating their follow-up [22, 23, 25]. In these studies, baseline biomarker levels were able to predict a clinical benefit from treatment with metronomic vinorelbine. If the correlations are confirmed, these biomarkers will be explored as covariates of model parameters related to efficiency.

A limitation of including both MPM and metastatic NSCLC in this phase I study is that results on efficacy parameters may depend on the proportion of each disease present.

The results obtained in this phase IA/IB should provide the rationale for a phase II, randomized study comparing a standard strategy versus a metronomic oral vinorelbine strategy based on mathematical modelling. The design of this trial can be viewed as a substantial effort. However, if successful, this approach paves the way for improved phase I designs applicable to other drugs across a wide range of diseases.

## Abbreviations

ALT: Alanine transaminase
ASCO: American society of clinical oncology
AST: Aspartate transaminase
CCTIRS: Comité Consultatif sur le Traitement de l’Information en Matière de Recherche dans le Domaine de la Santé (French : Advisory Committee on Information Processing in Material Research in the Field of Health)
CEC: Circulating endothelial cell
CNIL: Commission Nationale de l’Informatique et des Libertés (French: National Commission for Computing and Liberties)
CPP: Comité de protection des personnes (French: Committee to Protect People)
CT: Computerized tomography
CTC: Common toxicity criteria
DCR: Disease control rate
DDC: Duration of disease control
DR: Duration of response
ECOG: Eastern cooperative oncology group
ESMO: European society for medical oncology
QoL: Quality of life
FGF2: Fibroblast growth factor 2
IL8: Interleukin-8
LCSS: Lung cancer symptom scale
MPM: Malignant pleural mesothelioma
MTD: Maximum tolerated dose
NSCLC: Non-small cell lung cancer
ORR: Overall response rate
OS: Overall survival
OVTP: Optimal vinorelbine theoretical protocol
PD: Pharmacodynamics
PFS: Progression-free survival
PK: Pharmacokinetics
PK-PD: Pharmacokinetic-pharmacodynamic
PS: Performans status
RECIST: Response evaluation criteria in solid tumours
Tie2-TEK: Tyrosine kinase, endothelial
TIW: Thrice a week
TSP1: Thrombospondin-1
ULN: Upper limit of normal
VEGF: Vascular endothelial growth factor
VTP: Vinorelbine theoretical protocol
